# R0 Resection improves survival after multivisceral resection for pancreatic ductal adenocarcinoma

**DOI:** 10.1016/j.isci.2026.117352

**Published:** 2026-09-01

**Authors:** Artur Rebelo, Bodil Andersson, Samik Kumar Bandyopadhyay, Paulina Bereza-Carlson, Frederik Berrevoet, Bergthor Björnsson, Stefan Bouwense, Erik Bergquist, Florian Bösch, Markus Büchler, Nikolaos Chatzizacharias, Laurent Coubeau, Marie Crede, Cristine B. Pathirannehalage Don, Pieter Dries, Mert Erkan, Matthäus Felsenstein, Isabella Frigerio, Alessandro Giardino, Tim Glowka, Parsa Hadesi, Vera Hartman, Karin Johansen, Marie Klein, Johannes Klose, Karl Knipper, Carl-Stephan Leonhardt, Martin Loos, Thomas Malinka, Giovanni Marchegiani, Christopher Månsson, Riccardo Pellegrini, Giampaolo Perri, L.H. Poelsler, Geert Roeyen, Michael Rousek, Pablo Sancho, Stina Schild-Suhren, Thomas Schmidt, Leyre Serrablo, Alejandro Serrablo, Andrew Malvern Smith, Gregor A. Stavrou, Oliver Strobel, Shailesh Shrikhande, Alexandra Strobel, Ignazio Tarantino, Jozef Urdzik, Tim Vilz, Roberta Vella, Johanna Wennerblom, Patricia Wyzlic, Jörg Kleeff, Multivisceral Pancreatic Resections Research Group: A Collaboration of the European-African Hepato-Pancreato-Biliary Association (E-AHPBA)

**Affiliations:** 1 Department of Visceral, Vascular and Endocrine Surgery, University Hospital Halle (Saale), Martin-Luther-University Halle-Wittenberg, Halle, Germany; 2 Department of Clinical Sciences, Lund University, Lund, Sweden; 3 Manchester Royal Infirmary, Oxford Road, Manchester M13 9WL, UK; 4 Department of General and HPB Surgery and Liver Transplantation University Hospital Gent, Gent, Belgium; 5 Department of Surgery in Linköping and Department of Biomedical and Clinical Sciences, Linköping University, Linköping, Sweden; 6 Department of Surgery, Maastricht University Medical Centre, Maastricht 6229 HX, the Netherlands; 7 Department of General, Visceral and Pediatric Surgery, University Medical Center Göttingen, Göttingen-Weende, Germany; 8 Botton-Champalimaud Pancreatic Cancer Center, Lisbon, Portugal; 9 HPB and Liver Transplant Unit, Queen Elizabeth Hospital Birmingham, Birmingham, UK; 10 Department of Abdominal Surgery and Transplantation at Cliniques Universitaires Saint-Luc, Brussels, Belgium; 11 HPB and Liver Transplant Unit, Queen Elizabeth Hospital Birmingham, Birmingham, UK; 12 Department of Surgery, Charité – Universitätsmedizin Berlin, Berlin, Germany; 13 HPB Unit, Peschiera del Garda, Verona, Italy; 14 HPB Unit, Pederzoli Hospital, Verona, Italy; 15 Department of Surgery, Bonn University, Bonn, Germany; 16 Department of Surgery, Institute of Clinical Sciences, Sahlgrenska Academy, University of Gothenburg, Göteborg, Sweden; 17 Antwerp University Hospital, Department of HPB, Endocrine and Transplantation Surgery, Edegem, Belgium; 18 Department of General, Visceral, Thoracic and Transplantation Surgery, University Hospital of Cologne, Lindenthal, Germany; 19 Department of General Surgery, Division of Visceral Surgery, Medical University of Vienna, Wien, Austria; 20 Department of General, Visceral and Transplantation Surgery, Heidelberg University Hospital, Heidelberg, Germany; 21 University of Padua, Department of Surgical, Oncological and Gastroenterological Sciences, Padova, Italy; 22 Department of Surgical Sciences, Uppsala University, Uppsala, Sweden; 23 Department of Visceral, Transplant and Thoracic Surgery, Innsbruck, Austria; 24 Department of Surgery, Second Faculty of Medicine of Charles University and Military University Hospital, Prague, Czech Republic; 25 HPB Surgical Division, Miguel Servet University Hospital, Zaragoza, Spain; 26 St James University Hospital, Leeds, UK; 27 Department of General, Visceral and Thoracic Surgery, Surgical Oncology, Klinikum Saarbruecken, Saarbruecken, Germany; 28 Institute of Medical Epidemiology, Biostatistics, and Informatics, Interdisciplinary Center for Health Sciences, Medical Faculty of Martin Luther University Halle-Wittenberg, Wittenberg, Germany; 29 Department of General, Visceral, Endocrine, and Transplant Surgery, Cantonal Hospital of St. Gallen, St. Gallen, Switzerland; 30 NUTRIM School of Nutrition and Translational Research in Metabolism, Faculty of Health, Medicine and Life Sciences, Maastricht University, Maastricht, the Netherlands; 31 Department of Surgery, Sahlgrenska University Hospital, Region Västra Götaland, Gothenburg, Sweden; 32 Collegium Medicum, SAN University, Łódź, Poland; 33 Institute of Health, Midwifery and Nursing Science, Medical Faculty of Martin Luther University Halle-Wittenberg, University Medicine Halle, Halle (Saale), Germany; 34 Tata Memorial Hospital, Mumbai, India; 35 Mehmet Ali Aydinlar Acibadem University, Atakent University Hospital, Istanbul 34303, Turkey; 36 Department of Clinical Science, Intervention and Technology (CLINTEC), Karolinska Institutet, Stockholm, Sweden; 37 Division of Hepatobiliary and Pancreatic Surgery, Skåne University Hospital, Lund, Sweden; 38 Institute of Clinical Sciences, University of Birmingham, Birmingham, UK

**Keywords:** multivisceral, surgery, pancreatic

## Abstract

Achieving negative resection margins (R0) is central to curative surgery for pancreatic ductal adenocarcinoma (PDAC), but its prognostic relevance in multivisceral resections has been unclear. Using the largest international registry of multivisceral pancreatic resections, we performed a propensity score-matched analysis comparing R0 and R1 resections in patients undergoing multivisceral resection for PDAC. After 1:1 matching on pre- and intra-operative covariates, 222 patients were analyzed (111 R0, 111 R1). Perioperative morbidity, 90-day mortality, and intraoperative complications were comparable between groups. Median overall survival was 22.3 months after R0 resection versus 14.5 months after R1 resection; R1 status remained independently associated with poorer survival after adjustment for residual imbalances (hazard ratio [HR] 1.45, 95% confidence interval [CI] 1.02–2.07). Margin-negative resection is associated with improved long-term survival in multivisceral pancreatic surgery without an accompanying increase in perioperative risk, supporting R0 resection as a goal of surgical strategy at specialized centers.

## Highlights

• Largest propensity-matched study of R0 vs. R1 margins in multivisceral PDAC surgery
• 222 matched patients: R0 resection did not raise perioperative morbidity or mortality
• R0 resection gave longer survival: 22.3 vs. 14.5 months median (HR 1.45, *p* = 0.038)
• R0 remained an independent survival predictor after adjustment for residual imbalance

## Graphical abstract

**Figure:**
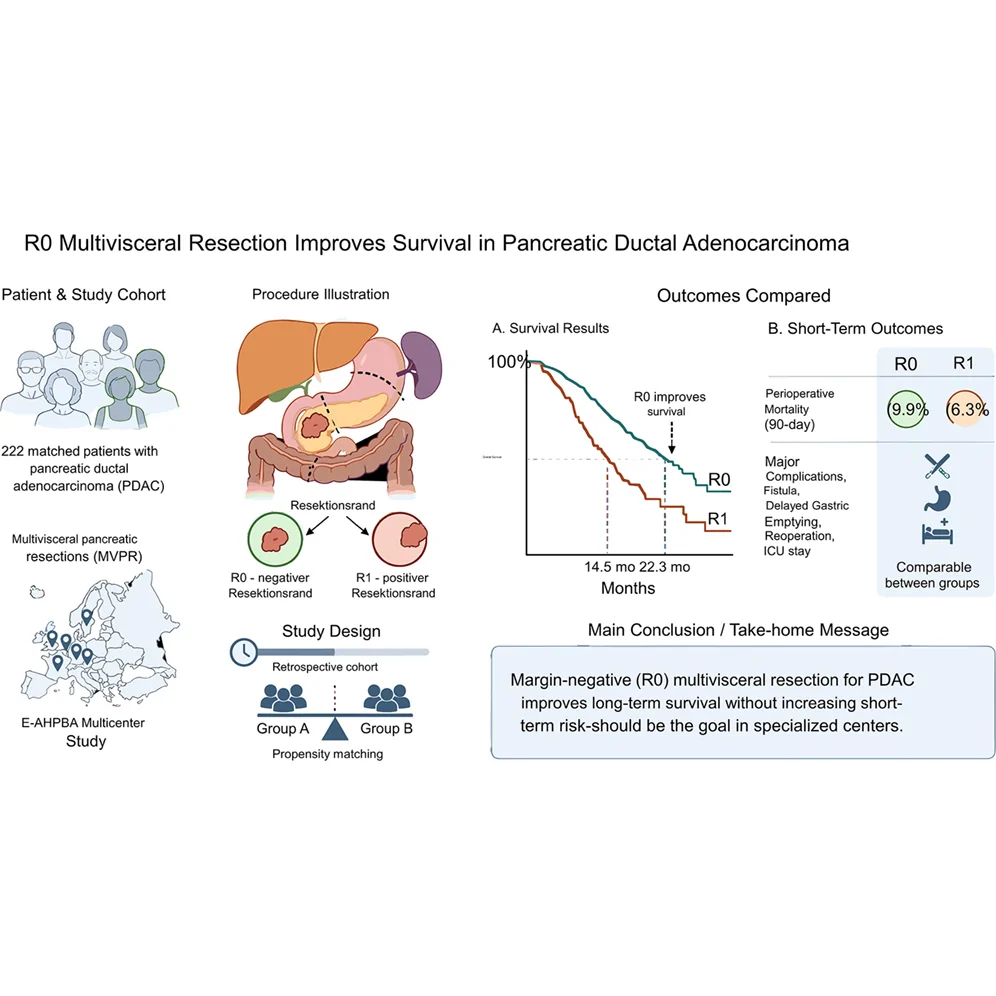

## Teaser

Medicine; Surgery; Oncology

## Introduction

The 5-year relative survival rate for pancreatic cancer remains low at approximately 11%.^1^ Projections from the International Agency for Research on Cancer estimate a 70% increase in new annual cases and a 72% rise in pancreatic cancer-related deaths, over the same period.^2^

In the surgical treatment of locally advanced pancreatic tumors, achieving complete tumor clearance often necessitates *en bloc* resections involving adjacent organs and major vascular structures.

The most frequent scenario involves pancreatic ductal adenocarcinoma (PDAC), which commonly infiltrates surrounding organs. Additional indications include locally advanced malignancies such as gastric cancer, gastrointestinal stromal tumors (GISTs), colorectal cancer with direct pancreatic invasion, and retroperitoneal sarcomas affecting the pancreatic region. In these complex cases, extended resections are undertaken with the goal of achieving complete (R0) tumor clearance, the only potentially curative approach. The prognostic impact of resection margin status in patients undergoing multivisceral oncologic resections for pancreatic ductal adenocarcinoma remains insufficiently defined, particularly with regard to perioperative risks and long-term survival.^3^^,^^4^^,^^5^

Multivisceral resection (MVR) is often required to achieve tumor clearance when PDAC invades adjacent organs, but has historically been associated with greater perioperative risk than standard pancreatectomy.^3^ In the largest international cohort reported to date, our group recently defined contemporary short-term outcome benchmarks for MVR across a range of tumor entities, identifying ASA class, surgical approach, and extent of resection as key predictors of morbidity and mortality^6^; however, that analysis did not evaluate long-term survival or the prognostic relevance of margin status. Whether the survival benefit conferred by R0 resection in standard pancreatectomy persists after MVR for PDAC remains unclear.^4^

To address this, the present study uses the same international registry to examine the association between resection margin status and both perioperative and long-term outcomes after MVR for PDAC, in what is, to our knowledge, the largest propensity score-matched analysis of this question to date.

The primary objective is to assess overall survival in patients with R0 versus R1 resections. The secondary objectives include 90-day mortality, perioperative complications, reoperation rates, intraoperative events, and ICU length of stay. By clarifying the oncological and surgical significance of margin status in multivisceral PDAC surgery, the study aims to inform patient selection, guide surgical strategy, and support evidence-based decision-making to improve outcomes.

## Results

### Patient characteristics and propensity score matching

This retrospective, multicenter cohort study represents the largest global database to date on multivisceral resections involving the pancreas and includes 1,440 patients, of whom 703 PDAC. Patients’ characteristics are presented in Table 1. For PS matching, only patients with R0 and R1 resection and without missing values in the matched covariates are analyzed; hence, those patients’ characteristics before (*n* = 391) and after (*n* = 222) matching are presented separately in Table 2. After matching, most standardized mean difference (SMD) for the covariates were below 0.1 (see the supplemental information, Figures S1–S3). Exceptions are the covariates Eastern Cooperative Oncology Group (ECOG) and tumor stage. Hence, the multivariable Cox regression was adjusted for these variables in order to address residual confounding. Moreover, all z-differences of matched covariates lie within a range of ±1.96, indicating good balance after PS matching (see the supplemental information, Table S1). The *SSQ*_*zDiff*_ reached a value of 1.27 which also indicated good global balance after PS matching.^7^ Before propensity score matching, 391 patients were included, of whom 274 (70.1%) underwent R0 resection, and 117 (29.9%) had an R1 resection. Patients in the R1 group tended to be older (71.7 vs. 64.6 years) and slightly more comorbid, although differences in baseline characteristics were small (absolute SMDs < 0.2 for all variables). After matching, 222 patients were included (111 R0 vs. 111 R1 resections), and all covariates were well balanced between groups (SMD ≈ 0.0 for all parameters), indicating successful matching. Age, sex distribution, body mass index (BMI), Charlson Comorbidity Index (CCI), American Society of Anesthesiologists (ASA) score, and ECOG performance status were comparable between matched cohorts. The distribution of tumor stage, resection type (pancreaticoduodenectomy, distal, or total pancreatectomy), and number of resected organs showed no relevant differences after matching. (Table 2).

**Table 1:** Patient characteristics for overall cohort

	Overall (*N* = 703)
**Age (years)**	

Mean (SD)	66.1 (26.6)
Missing	6 (0.9%)

**Gender**	

Male	399 (56.8%)
Female	303 (43.1%)
Missing	1 (0.1%)

**BMI**	

Mean (SD)	24.5 (4.58)
Missing	72 (10.2%)

**CCI**	

Mean (SD)	0.936 (1.14)
Missing	126 (17.9%)

**Resection margins**	

R0	387 (55.0%)
R1	170 (24.2%)
R2	7 (1.0%)
Missing	139 (19.8%)

**ASA**	

1	90 (12.8%)
2	317 (45.1%)
3	241 (34.3%)
4	11 (1.6%)
Missing	44 (6.3%)

**ECOG**	

1	285 (40.5%)
2	257 (36.6%)
3	38 (5.4%)
4	10 (1.4%)
Missing	113 (16.1%)

**Type of resection**	

TP	90 (12.8%)
DP	459 (65.3%)
PD	154 (21.9%)

**Tumor stage**	

I	59 (8.4%)
II	226 (32.1%)
III	298 (42.4%)
IV	87 (12.4%)
Missing	33 (4.7%)

**Number of resected organs**	

1	386 (54.9%)
2	202 (28.7%)
3	77 (11.0%)
4+	38 (5.4%)

**Adjuvant radiotherapy**	

Yes	25 (3.6%)
No	480 (68.3%)
Missing	198 (28.2%)

**Neoadjuvant radiotherapy**	

Yes	15 (2.1%)
No	673 (95.7%)
Missing	15 (2.1%)

**Adjuvant chemotherapy**	

Yes	339 (48.2%)
No	156 (22.2%)
Missing	208 (29.6%)

**Neoadjuvant chemotherapy**	

Yes	229 (32.6%)
No	469 (66.7%)
Missing	5 (0.7%)

*N* = 1,440 overall; PDAC subgroup (*n* = 703). Values are presented as *n* (%) or mean (SD).

**Table 2:** Patients characteristics before and after PS matching

	Overall (*N* = 391)	Before matching			After matching			
		R0 Resection (*N* = 274)	R1 Resection (*N* = 117)	SMD	Overall (*N* = 222)	R0 Resection (*N* = 111)	R1 Resection (*N* = 111)	SMD
Age	66.7 (34.3)	64.6 (10.6)	71.7 (60.5)	−0.117	65.8 (10.5)	65.7 (10.4)	65.8 (10.7)	−0.001
Gender	–	–	–	0.064	–	–	–	0.018
Male	216 (55.2%)	154 (56.2%)	62 (53.0%)	–	119 (53.6%)	60 (54.1%)	59 (53.2%)	–
Female	175 (44.8%)	120 (43.8%)	55 (47.0%)	–	103 (46.4%)	51 (45.9%)	52 (46.8%)	–
BMI	24.5 (4.14)	24.7 (4.20)	24.1 (3.98)	0.134	24.1 (4.00)	24.1 (3.98)	24.1 (4.04)	−0.006
CCI	0.923 (1.11)	0.916 (1.10)	0.940 (1.14)	−0.021	0.923 (1.06)	0.910 (1.00)	0.937 (1.12)	−0.024

**ASA Score**								

1	55 (14.1%)	35 (12.8%)	20 (17.1%)	−0.115	35 (15.8%)	16 (14.4%)	19 (17.1%)	−0.072
2	189 (48.3%)	141 (51.5%)	48 (41.0%)	0.212	99 (44.6%)	51 (45.9%)	48 (43.2%)	0.055
3	138 (35.3%)	92 (33.6%)	46 (39.3%)	−0.118	82 (36.9%)	41 (36.9%)	41 (36.9%)	0.000
4	9 (2.3%)	6 (2.2%)	3 (2.6%)	−0.024	6 (2.7%)	3 (2.7%)	3 (2.7%)	0.000

**ECOG**								

1	171 (43.7%)	115 (42.0%)	56 (47.9%)	−0.118	108 (48.6%)	57 (51.4%)	51 (45.9%)	0.108
2	202 (51.7%)	149 (54.4%)	53 (45.3%)	0.182	102 (45.9%)	49 (44.1%)	53 (47.7%)	−0.072
3	16 (4.1%)	8 (2.9%)	8 (6.8%)	−0.155	12 (5.4%)	5 (4.5%)	7 (6.3%)	−0.071
4	2 (0.5%)	2 (0.7%)	0 (0.0%)	0.102	0 (0.0%)	0 (0.0%)	0 (0.0%)	0.000

**Type of resection**								

TP	57 (14.6%)	34 (12.4%)	23 (19.7%)	−0.182	40 (18.0%)	21 (18.9%)	19 (17.1%)	0.045
DP	251 (64.2%)	181 (66.1%)	70 (59.8%)	0.127	135 (60.8%)	65 (58.6%)	70 (63.1%)	−0.092
PD	83 (21.2%)	59 (21.5%)	24 (20.5%)	0.025	47 (21.2%)	25 (22.5%)	22 (19.8%)	0.067

**Tumor stage**								

I	28 (7.2%)	22 (8.0%)	6 (5.1%)	0.132	12 (5.4%)	6 (5.4%)	6 (5.4%)	0.000
II	129 (33.0%)	92 (33.6%)	37 (31.6%)	0.042	71 (32.0%)	35 (31.5%)	36 (32.4%)	−0.019
III	173 (44.2%)	117 (42.7%)	56 (47.9%)	−0.103	103 (46.4%)	50 (45.0%)	53 (47.7%)	−0.054
IV	61 (15.6%)	43 (15.7%)	18 (15.4%)	0.009	36 (16.2%)	20 (18.0%)	16 (14.4%)	0.100

**Number of resected organs**								

1	210 (53.7%)	147 (53.6%)	63 (53.8%)	−0.004	121 (54.5%)	62 (55.9%)	59 (53.2%)	0.054
2	119 (30.4%)	86 (31.4%)	33 (28.2%)	0.071	63 (28.4%)	31 (27.9%)	32 (28.8%)	−0.020
3	45 (11.5%)	29 (10.6%)	16 (13.7%)	−0.090	28 (12.6%)	13 (11.7%)	15 (13.5%)	−0.052
4+	17 (4.3%)	12 (4.4%)	5 (4.3%)	−0.005	10 (4.5%)	5 (4.5%)	5 (4.5%)	0.000

Before matching, *n* = 391 (R0: *n* = 274; R1: *n* = 117); after 1:1 propensity score matching, *n* = 222 (R0: *n* = 111; R1: *n* = 111). Values are presented as *n* (%) or mean (SD). SMD, standardized mean difference (absolute SMD < 0.1 indicates good covariate balance).

### Perioperative outcomes

Table 3 summarizes perioperative outcomes for the matched cohort. Intraoperative characteristics were comparable between groups. Mean blood loss was 863 mL for R0 and 867 mL for R1 resections, and mean operative duration was 342 in both groups. Intraoperative complications occurred in 23.4% of R0 and 30.6% of R1 cases. Reoperation rates were low (17.1% vs. 12.6%). Postoperative morbidity did not differ significantly between margin groups. The incidence of postoperative bleeding was similar (19.8% vs. 16.2%), while clinically relevant pancreatic fistula occurred in 27.9% (R0) and 31.5% (R1). Delayed gastric emptying was observed in 16.2% (R0) and 18.9% (R1) of patients. According to the Clavien-Dindo classification, severe (grades III–V) complications occurred in 37.8% (R0) and 36.9% (R1), with comparable distributions across grades. The mean ICU stay was approximately 3–4 days in both groups, and 90-day mortality was 9.9% for R0 and 6.3% for R1 resections.

**Table 3:** Outcomes for the overall cohort

	R0 Resection (*N* = 111)	R1 Resection (*N* = 111)	Overall (*N* = 222)
**Blood loss (mL)**			

Mean (SD)	863 (1,380)	867 (892)	865 (1,180)
Missing	35 (31.5%)	46 (41.4%)	81 (36.5%)

**Duration of surgery (min)**			

Mean (SD)	342 (149)	342 (126)	342 (138)
Missing	11 (9.9%)	7 (6.3%)	18 (8.1%)

**Intraoperative complications**			

No	85 (76.6%)	77 (69.4%)	162 (73.0%)
Yes	26 (23.4%)	34 (30.6%)	60 (27.0%)

**Reoperation**			

Yes	19 (17.1%)	14 (12.6%)	33 (14.9%)
No	76 (68.5%)	84 (75.7%)	160 (72.1%)
Missing	16 (14.4%)	13 (11.7%)	29 (13.1%)

**Bleeding**			

No	89 (80.2%)	93 (83.8%)	182 (82.0%)
Yes	22 (19.8%)	18 (16.2%)	40 (18.0%)

**Fistula**			

No	80 (72.1%)	76 (68.5%)	156 (70.3%)
Yes	31 (27.9%)	35 (31.5%)	66 (29.7%)

**DGE**			

No	93 (83.8%)	90 (81.1%)	183 (82.4%)
Yes	18 (16.2%)	21 (18.9%)	39 (17.6%)

**CD complications**			

None	23 (20.7%)	33 (29.7%)	56 (25.2%)
Grade I-II	46 (41.4%)	37 (33.3%)	83 (37.4%)
Grade III-V	42 (37.8%)	41 (36.9%)	83 (37.4%)

**Duration of ICU stay (days)**			

Mean (SD)	4.43 (6.10)	3.28 (6.10)	3.84 (6.11)
Missing	17 (15.3%)	12 (10.8%)	29 (13.1%)

**90-day mortality**			

Yes	11 (9.9%)	7 (6.3%)	18 (8.1%)
No	95 (85.6%)	99 (89.2%)	194 (87.4%)
Missing	5 (4.5%)	5 (4.5%)	10 (4.5%)

Values are presented as *n* (%) or mean (SD); matched cohort, *N* = 222 (R0: *n* = 111; R1: *n* = 111). Given the matched design, formal between-group significance testing was not performed for these descriptive comparisons.

### Survival outcomes: Kaplan-Meier survival analysis

Valid survival data were available for 206 patients. The median overall survival of the cohort was 17.6 months (95% confidence interval [CI], 14.6–22.4). Patients with R0 resection had a longer median survival of 22.3 months (95% CI, 17.8–29.6) compared with 14.5 months (95% CI, 11.7–19.1) after R1 resection. The corresponding 1-, 3-, and 5-year survival rates were 63.8%, 28.3%, and 18.2% for the overall cohort; 69.7%, 32.7%, and 21.6% for R0 resections; and 57.4%, 23.1%, and 14.5% for R1 resections, respectively. Overall, patients with R0 resection demonstrated a consistent survival advantage across all time points (Table 4).

**Table 4:** Median-survival and 1-, 3-, and 5-year survival rates

	Overall	R0 Resection	R1 Resection
Median survival time	17.6 months 95% CI (14.6–22.4)	22.3 months 95% CI (17.8–29.6)	14.5 months 95% CI (11.7–19.1)
1-year survival rate	63.8% 95% CI (57.2%–71.3%)	69.7% 95% CI (61.1%–79.6%)	57.4% 95% CI (47.7%–69.0%)
3-year survival rate	28.3% 95% CI (21.9%–36.5%)	32.7% 95% CI (24.1%–44.4%)	23.1% 95% CI (14.9%–36.0%)
5-year survival rate	18.2% 95% CI (12.6%–26.4%)	21.6% 95% CI (13.9%–33.6%)	14.5% 95% CI (7.6%–27.7%)

Kaplan-Meier method; values are presented as % (95% CI) unless otherwise stated. *N* = 206 patients with evaluable survival data (of 222 matched patients).

**Figure 1:**
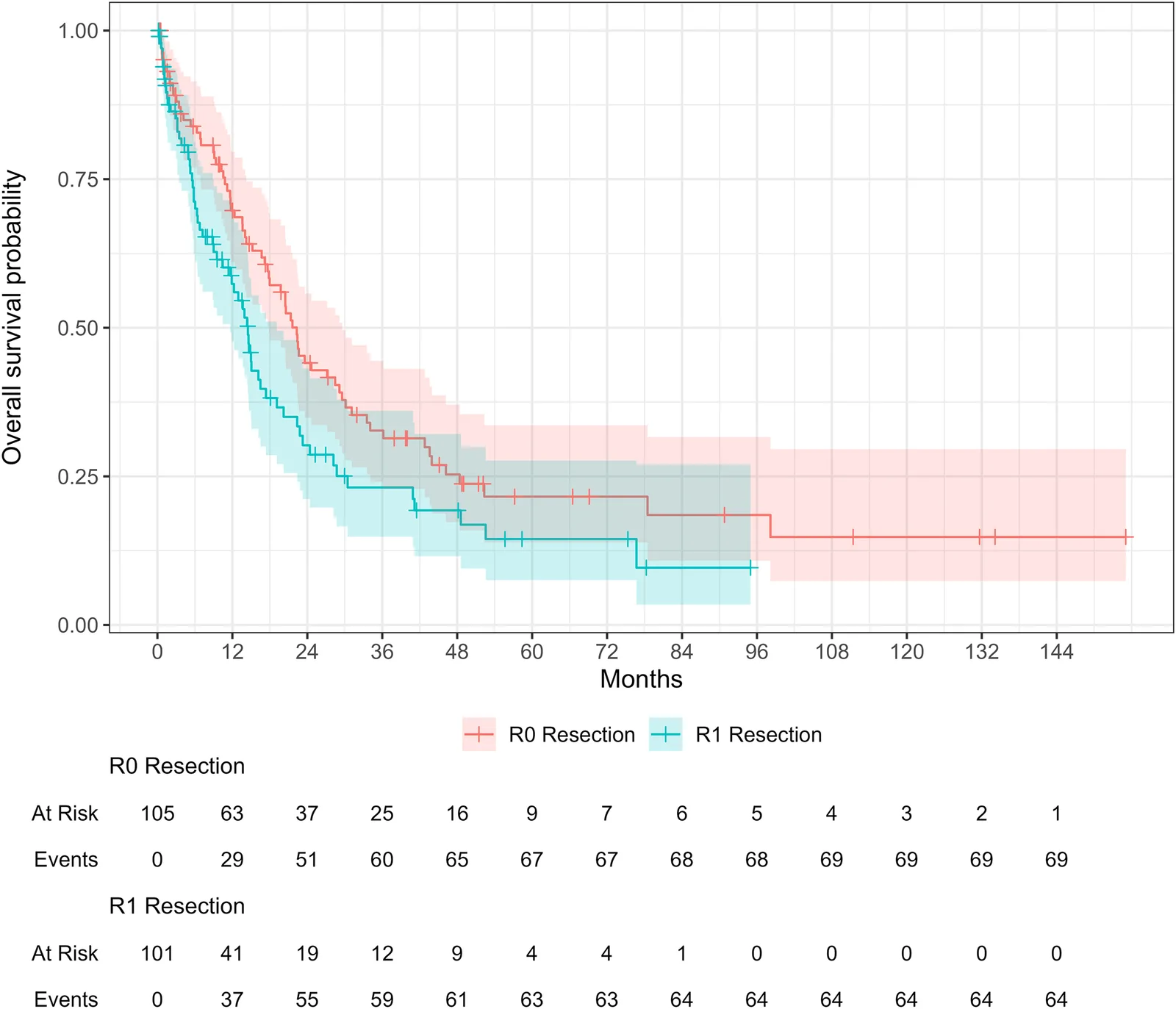
Kaplan-Meier survival analysis Kaplan-Meier overall survival curves for R0 versus R1 resection in the propensity score-matched cohort (*N* = 206 with evaluable survival data); shaded bands represent 95% confidence intervals; survival difference assessed by log rank test (see Table 5 for the corresponding univariable Cox regression hazard ratio).

Figure 1 illustrates the Kaplan-Meier survival curves for patients with R0 and R1 resections after matching. Patients with R0 resection demonstrated a visibly improved overall survival compared with those with R1 resection. In the univariable Cox regression analysis (Table 5), R1 resection was associated with a trend toward worse overall survival compared with R0 resection (hazard ratio [HR], 1.47; 95% CI, 1.04–2.07; *p* = 0.030). After adjustment for residual covariate imbalances in the multivariable Cox regression model (Table 6), R1 resection remained an independent predictor of reduced overall survival (HR, 1.45; 95% CI, 1.02–2.07; *p* = 0.038). None of the other covariates showed a statistically significant association with survival. The results of the sensitivity analyses were consistent with the primary propensity score analyses. Both the conventional univariable and multivariable Cox proportional hazards models performed in the overall cohort and the analyses based on multiple imputation of missing data yielded comparable effect estimates without relevant deviations in the direction or magnitude of the associations. Overall, these findings support the robustness of the primary results and suggest that the observed associations were not materially influenced by the analytical approach or by missing data.

**Table 5:** Univariable cox regression

Characteristic	HR^a^	95% CI^a^	*p* value
**Resection margin**			

R0 Resection	Ref.	–	–
R1 Resection	1.47	1.04, 2.07	**0.030**

Cox proportional hazards regression; matched cohort, *N* = 206 with evaluable survival data. Bold indicates *p* values under < 0.05.

a HR, hazard ratio; CI, confidence interval.

**Table 6:** Multivariable Cox Regression (adjusted for covariates that are still imbalanced after PS matching)

Characteristic	HR^a^	95% CI^a^	*p* value
**Resection margin**			

R0 Resection	Ref.	–	–
R1 Resection	1.45	1.02, 2.07	**0.038**

**ECOG**			

1	Ref-	–	–
2	1.14	0.80, 1.62	0.5
3	1.11	0.50, 2.44	0.8

**Tumor stage**			

1	Ref.	–	–
2	0.91	0.41, 2.05	0.8
3	0.90	0.41, 1.96	0.8
4	0.71	0.29, 1.75	0.5

Cox proportional hazards regression adjusted for covariates that remained imbalanced after propensity score matching (ECOG performance status, tumor stage); matched cohort, *N* = 206 with evaluable survival data. Bold indicates *p* values under < 0.05.

a HR, hazard ratio; CI, confidence interval.

## Discussion

In this large, multicenter cohort of patients undergoing multivisceral pancreatic resections for PDAC, resection margin status was associated with long-term survival. While perioperative morbidity and short-term mortality were comparable between R0 and R1 resections, patients with R0 margins experienced significantly longer overall survival, with higher 1-, 3-, and 5-year survival rates. In multivariable analysis, R1 resection remained independently associated with poorer survival after adjustment for residual covariate imbalances; none of the other covariates showed a statistically significant association.

These findings support the oncologic value of achieving margin-negative resections, even in the setting of technically complex multivisceral procedures. Multiple studies have reaffirmed that long-term survival in pancreatic cancer is strongly influenced by multimodal treatment strategies.^8^^,^^9^^,^^10^^,^^11^ More recently, Stoop et al.^12^ demonstrated that the combination of neoadjuvant and adjuvant therapy can further prolong median survival to over 40 months, particularly when mFOLFIRINOX is used in the adjuvant setting. These findings collectively emphasize that aggressive surgical strategies, when embedded within structured multimodal treatment plans, can achieve meaningful long-term outcomes. Also, when evaluating systemic therapy in vulnerable populations, the GIANT trial^13^ showed no significant survival difference between gemcitabine and 5-FU/leucovorin in elderly patients with metastatic disease, highlighting the limited benefit of chemotherapy alone in frail populations and underscoring the critical need for individualized, stage-specific, and biology-driven treatment approaches.

The value of negative margin status has been further validated through meta-analyses. Strobel et al.^14^ and Leonhardt et al.^15^ showed that R0 resections are associated with significantly improved survival (HR, 1.50 and 1.77, respectively). Similarly, Ghaneh et al.^16^ demonstrated that direct margin involvement and even margins within 1 mm were associated with inferior survival, further reinforcing the principle that complete tumor clearance is paramount.

Nonetheless, the technical demands and risks of multivisceral resections should not be underestimated. Hartwig et al.^1^ reported significantly higher morbidity (27% vs. 17%) and mortality (7% vs. 2%) in patients undergoing extended left pancreatectomy, with ASA III–IV status, prolonged operative time, and blood transfusion emerging as predictors of adverse outcomes. These findings were corroborated by Brunner et al., highlighting the need for careful patient selection and perioperative optimization.^17^ A direct within-cohort comparison to patients who did not require adjacent organ resection was not feasible, as this registry exclusively enrolled patients undergoing multivisceral resection. Nonetheless, it is informative to contextualize our findings against published outcomes of standard (non-multivisceral) pancreatic resections: large series of R0 standard pancreatectomy reports a median overall survival of approximately 22–28 months. This is corroborated by a recent nationwide Dutch cohort of 5,553 resected PDAC patients, in which median overall survival increased across successive treatment eras to 23.6 months in the most recent period, with 5- and 10-year OS rates of 18.0% and 10.6%, respectively.^18^ The median OS of 22.3 months observed in our R0 multivisceral cohort falls within this range, suggesting that, when negative margins are achievable, multivisceral resection need not carry an inherent oncological penalty relative to standard resection, despite its greater technical complexity. These figures are further contextualized by real-world data from a centralized UK oncology service, in which structured perioperative pathways for resected PDAC achieved a 30-day mortality of 2.4% and an adjuvant chemotherapy completion rate of 82%, supporting the feasibility of achieving comparable safety and treatment-delivery benchmarks in appropriately selected multivisceral resection candidates.^19^

Improved survival in pancreatic cancer has been driven by multiple advances, including safer and more aggressive surgical techniques, enhanced perioperative care, and more effective multimodal treatment strategies. Achieving complete tumor clearance remains a cornerstone of curative intent, and in selected cases, this requires multivisceral resection to obtain R0 status. These extended resections, when performed in experienced centers, contribute significantly to long-term outcomes by maximizing the chances of margin-negative surgery.^20^^,^^21^

Historically, R0 resection rates have been lower in multivisceral resections compared with standard pancreatectomy, as shown in a meta-analysis that included 176 patients undergoing multivisceral resections and 713 standard cases, where R0 rates ranged from 55% to 57% and 61%–76%, respectively.^4^ In our study, the R0 rate of 53.6%,which was previously published, aligns with the upper range of prior multivisceral series, suggesting that complete tumor clearance is feasible with appropriate surgical expertise. While reduced R0 rates have often been viewed as a limitation of extended resections, our data indicate that margin-negative resection remains achievable, and those patients in whom margin-negative resection is achieved are observed to have longer survival. It should be emphasized that this association is observational rather than causal: achieving R0 margins may in part reflect less biologically aggressive or more favorably located tumors that are inherently easier to clear completely, rather than R0 status itself independently conferring a survival benefit. Positive margin (R1) status may therefore act, at least partly, as a marker of aggressive tumor biology or advanced local disease rather than as an independent, modifiable determinant of outcome. Definitively establishing a causal survival benefit of achieving R0 resection would require prospective, ideally randomized, data, which are difficult to generate in this surgical context.

In a recent randomized trial on locally advanced pancreatic cancer (LAPC), the median overall survival in both treatment arms, FOLFIRINOX and gemcitabine, was approximately 15 months (15.7 vs. 15.4 months). While FOLFIRINOX significantly improved progression-free survival, it did not confer a survival advantage over gemcitabine.^22^ Other trials showed resectability higher resectability rates in this context.^23^^,^^24^ In this study, only a small proportion of patients underwent surgical resection, underscoring the limited impact of systemic therapy alone on long-term survival in locally advanced pancreatic cancer. The overall survival of approximately 15 months reflects only a modest improvement compared with patients with metastatic disease, who typically achieve a median survival of around 11 months with chemotherapy alone.^25^ This highlights the pressing need to integrate surgical strategies, when feasible, into multimodal treatment concepts to achieve meaningful survival benefits. Notably, patients in our study who were able to undergo surgical resection achieved a median overall survival of 20.2 months, suggesting a potential survival benefit in selected cases. These findings highlight the need for future research to evaluate the role of neoadjuvant or adjuvant strategies in patients requiring multivisceral resections, with particular emphasis on improving resectability rates and achieving negative margins to optimize oncologic outcomes. In this context, the role of complex surgical approaches, including vascular and multivisceral resections, must be further investigated to determine their potential in extending survival and offering curative intent in carefully selected patients with locally advanced disease.

In this multicenter, propensity score-matched analysis of patients undergoing multivisceral pancreatic resections for PDAC, R0 resection margin status was independently associated with significantly improved long-term survival, without a corresponding increase in perioperative morbidity or mortality. These findings underscore the oncological value of achieving negative margins, even in the context of complex, extended resections. Future studies should further investigate the role of multimodal treatments with vascular and multivisceral resections to determine their potential in extending survival and offering curative intent in carefully selected patients with locally advanced disease.

### Limitations of the study

This study has several limitations that warrant consideration. (1) Retrospective design and selection bias. The retrospective design introduces inherent selection bias and limits causal inference regarding the relationship between resection margin status and outcomes; a prospective registry with predefined data collection would reduce this risk. (2) Missing data. Of the 703 PDAC patients in the overall registry, more than half were excluded from the propensity-score-matched analysis because of incomplete covariate data, leaving 391 patients eligible for matching. This substantial reduction is an important limitation; we addressed it with multivariable Cox regression in the full complete-case cohort and a multiple-imputation sensitivity analysis (supplemental information, Tables S2–S4), both of which yielded results consistent with the primary matched analysis, but future prospective registries should prioritize complete baseline data capture. (3) Residual confounding after matching. Although propensity score matching was used to minimize confounding, residual imbalances in covariates such as ECOG performance status and tumor stage persisted after matching and were adjusted for in multivariable analyses; unmeasured confounders (e.g., tumor biology, surgeon/center volume) cannot be excluded and would require prospective data with more granular covariate capture to address. (4) Heterogeneity in pathological margin assessment. Despite the multicenter nature of the study, variation in surgical technique and pathological assessment of resection margins across more than 30 participating centers, and potential changes in pathological examination practice over the 12-year study period, may have influenced margin classification and outcome measures; future multicenter studies should adopt a standardized, centrally reviewed pathology protocol for margin assessment. (5) Limited sample size with complete survival data. Valid survival data were available for only 206 of 222 matched patients, which may affect the precision of long-term survival estimates; larger prospective cohorts would improve precision. (6) No quality-of-life or functional outcome data. This study focused on survival and perioperative outcomes but did not assess long-term functional outcomes or quality of life, which are highly relevant in patients undergoing complex, multivisceral resections; future studies should incorporate patient-reported outcome measures. (7) No formal LAPC/borderline-resectable classification. The registry was not designed with a formal, contemporary categorization of borderline-resectable versus locally advanced disease, and such data were not systematically collected; future prospective registries should standardize resectability classification at baseline. (8) Variability in systemic therapy use over time. The 12-year study period (2010–2022) spans substantial evolution in systemic therapy for PDAC, and variability in adjuvant/neoadjuvant regimens used across this period and across centers was not fully captured, which may confound comparisons of long-term outcomes; future studies should account for era and regimen effects explicitly. (9) Sex and ethnicity reporting. Patient ethnicity was not systematically recorded across the more than 30 participating international centers and could therefore not be analyzed; sex was included as a covariate in propensity score matching, but no formal subgroup or interaction analysis of its association with outcomes was performed; future registries should capture these variables systematically to allow such analyses.

Future research should aim to validate these findings in prospective, multicenter settings with standardized margin definitions and pathological protocols. Incorporating patient-reported outcomes, as well as imaging and molecular biomarkers, may help further refine individualized treatment strategies.

## Resource availability

### Lead contact

Further information and requests for resources and reagents should be directed to and will be fulfilled by the lead contact, Artur Rebelo (artur.rebelo@uk-halle.de).

### Materials availability

This study did not generate new unique reagents or materials.

### Data and code availability

• De-identified patient-level data supporting the findings of this study are available from the corresponding author upon reasonable request, subject to the institutional data-sharing agreements and national data protection regulations of the participating centers.
• This study did not generate original code; statistical analyses were performed in RStudio (v4.4.2) using standard packages, as detailed in the STAR Methods (quantification and statistical analysis).
• Any additional information required to reanalyze the data reported in this paper is available from the lead contact upon request.

## Acknowledgments

The authors thank the local investigators, data managers, and administrative staff at all participating centers for their contribution to data collection and curation, and thank the European-African Hepato-Pancreato-Biliary Association (E-AHPBA) for endorsing and supporting this collaborative project.

## Author contributions

A.R., conceptualization, methodology, formal analysis, investigation, project administration, writing – original draft, and writing – review and editing; J.K., conceptualization, supervision, and writing – review and editing; A.S., methodology, formal analysis, software, validation, and writing – review and editing. All remaining co-authors (Multivisceral Pancreatic Resections Research Group) contributed to data curation, investigation, and resources at their respective centers, and reviewed and approved the final manuscript (writing – review and editing).

## Declaration of interests

The authors declare no competing interests.

## STAR★Methods

### Key resources table

**Table:** 

REAGENT or RESOURCE	SOURCE	IDENTIFIER
**Software and algorithms**		

Software: RStudio, v4.4.2	Posit Software, PBC	https://www.rstudio.com
Software: R, v ≥ 4.0	R Core Team	https://www.r-project.org
Data collection platform: REDCap	Vanderbilt University	https://www.project-redcap.org

### Experimental model and study participant details

This retrospective cohort study included adult patients (≥18 years) who underwent multivisceral pancreatic resection for histologically confirmed pancreatic ductal adenocarcinoma (PDAC) at participating high-volume hepato-pancreato-biliary centers worldwide between January 1, 2010 and December 31, 2022 (see Table 1 for age distribution). Patient sex was recorded as a binary covariate (male/female, as documented in institutional records) and was included as a covariate in propensity score matching; see limitations of the study for reporting constraints regarding sex- and ethnicity-related analyses. This study is not a registered clinical trial and no clinical trial registry number applies. Ethical approval for the overall project was granted by the Ethics Committee of the University Hospital Halle (Saale), Germany (protocol/processing number 2023-211), confirming that the study conforms to relevant regulatory and ethical standards; each participating center additionally secured national and local ethical clearance in accordance with institutional and national regulations. The study protocol was reviewed, approved, and endorsed by the European-African Hepato-Pancreato-Biliary Association (E-AHPBA). Given the retrospective, anonymised nature of the analysis, individual informed consent was waived where not otherwise mandated by local regulations.

### Method details

#### Study design and setting

This retrospective, multicenter, international cohort study investigates the outcomes of multivisceral pancreatic resections for PDAC. Data were collected from specialized surgical centers across the globe, focusing on patients who underwent such procedures between January 1, 2010, and December 31, 2022. Centers were selected based on their expertise in complex pancreatic surgery, and data were prospectively recorded by local investigators using a centralized REDCap database to ensure standardized collection across sites.

All study procedures adhered to local ethical and regulatory requirements. Where applicable, data were anonymized in accordance with national standards when individual patient consent was not mandated. Ethical approval for the overall project was granted by the Ethics Committee of the University Hospital Halle (Saale), Germany (processing number 2023-211). Each participating center also secured national and local ethical clearance in line with national and institutional regulations. The study protocol was formally reviewed, approved, and endorsed by the European-African Hepato-Pancreato-Biliary Association (E-AHPBA).

#### Participants

Inclusion Criteria:

• Patients who underwent elective multivisceral resections involving the pancreas for PDAC.
• Cases with curative intent for non-metastatic PDAC requiring en-bloc resection of adjacent organs.
• Availability of complete perioperative data, including demographic, surgical, and outcome measures.

Exclusion Criteria:

• Patients who underwent isolated pancreatic resection (without additional organ resection).
• Resections for other indications than PDAC.
• Palliative resections where curative intent was not pursued.
• Revision pancreatectomies and cases with pancreatic metastases from other primary tumors.

#### Intervention/Exposure

The study investigates patients who underwent multivisceral resections for PDAC, defined as pancreatic resection (total pancreatectomy, distal pancreatectomy, or pancreatoduodenectomy) combined with the resection of at least one additional organ, including: colon, stomach, adrenal gland, liver, kidney, small intestine and spleen. Portal vein or arterial resections, jejunal resection, or partial stomach resection performed as part of a standard pancreatoduodenectomy, as well as splenectomy conducted during a standard distal pancreatectomy, were not classified as multivisceral resections.

#### Outcomes

Primary Outcome:

• Overall Survival (OS) - Measured from the date of surgery to death from any cause or last follow-up.

Secondary Outcomes:

• 90-day postoperative mortality.
• Perioperative morbidity (Clavien-Dindo classification^26^).
• Surgical complications, including pancreatic fistula, postoperative bleeding, and delayed gastric emptying (ISGPS definitions^27^^,^^28^^,^^29^).
• Reoperation rates and intraoperative adverse events based on Satava classification.^30^
• Resection margin status (R0, R1, R2) based on the Royal College of Pathologists definition.^31^
• Intensive care unit (ICU) (days) and hospital length of stay (days).

### Quantification and statistical analysis

Demographic and operative characteristics were summarized using appropriate descriptive summary measures. We used propensity score (PS) matching to estimate the average treatment effect of treated (target estimand: ATT) regarding resection margins. Therefore, a 1:1-nearest neighbor procedure without replacement with a caliper of width, equal to 0.2 of the standard deviation of the logit of the PS was applied. The PS was estimated using logistic regression with the following covariates: age, gender, Body Mass Index (BMI), Charlson Comorbidity Index (CCI), American Society of Anesthesiologists (ASA) physical status, and Eastern Cooperative Oncology Group (ECOG) performance status, type of resection, tumor stage, and number of resected organs. These covariates were chosen due to empirical knowledge and clinical relevance. For balance diagnostic before and after matching, standardized mean differences (SMD) and z-differences^32^ were estimated and graphically illustrated. In addition, the overall balance was evaluated using sum of squared z-differences (*SSQ*_*zDiff*_).^7^^,^^33^ After matching, various outcomes, including mortality and complications were compared between the matched groups. Overall survival from surgery was measured from the date of surgery to either death from any cause or the last follow-up. Median survival was estimated using the Kaplan–Meier method, and survival differences between groups were assessed with the log rank test. Univariate and multivariate analyses were conducted using the Cox proportional hazards model, with results reported as hazard ratios (HR) and 95%-confidence intervals (CI). The multivariable Cox model was adjusted for covariates that remained unbalanced after matching. In addition to the propensity score analyses, several sensitivity analyses were performed to evaluate the robustness of the findings. First, univariable and multivariable Cox proportional hazards regression models were fitted using the complete study cohort. Second, missing covariate data were handled using multiple imputation by chained equations (MICE). Twenty imputed datasets (m = 20) were generated using an iterative chained-equations approach, with predictive mean matching applied to continuous variables and logistic or multinomial logistic regression models applied to categorical variables, as appropriate. Each imputed dataset was analyzed separately using the prespecified Cox proportional hazards model, and the resulting regression coefficients and standard errors were combined according to Rubin’s rules to obtain pooled HRs, 95% CIs, and corresponding *p*-values. A *p*-value of less than 0.05 was considered statistically significant, however all results are of exploratory nature. Statistical analyses were performed using RStudio Version 4.4.2. This study is reported in accordance with the STROBE (Strengthening the Reporting of Observational Studies in Epidemiology) guideline for cohort studies (completed checklist provided as a Supplementary File).

Statistical tests, exact sample sizes, and effect estimates for each comparison are reported directly in the corresponding figure and table legends (log rank test for the Kaplan–Meier comparison in Figure 1; univariable and multivariable Cox proportional hazards regression in Tables 5 and 6). Full details of the statistical approach are given below.

## Supplemental information

Document S1. Figures S1–S3 and Tables S1–S4

Data S1. Completed STROBE checklist for reporting of this observational cohort study
