# Supplementary material for: R0 Resection improves survival after multivisceral resection for pancreatic ductal adenocarcinoma
Source: iScience. 2026 Sep 1;29(9):117352. doi: 10.1016/j.isci.2026.117352 (PMC13571119; doi:10.1016/j.isci.2026.117352)
Supplement: Supplementary file 1 [file mmc1.pdf]

iScience, Volume *29*

Supplemental information

R0 Resection improves survival after multivisceral resection for pancreatic ductal adenocarcinoma

Artur Rebelo, Bodil Andersson, Samik Kumar Bandyopadhyay, Paulina Bereza-Carlson, Frederik Berrevoet, Bergthor Björnsson, Stefan Bouwense, Erik Bergquist, Florian Bösch, Markus Büchler, Nikolaos Chatzizacharias, Laurent Coubeau, Marie Crede, Cristine B. Pathirannehalage Don, Pieter Dries, Mert Erkan, Matthäus Felsenstein, Isabella Frigerio, Alessandro Giardino, Tim Glowka, Parsa Hadesi, Vera Hartman, Karin Johansen, Marie Klein, Johannes Klose, Karl Knipper, Carl-Stephan Leonhardt, Martin Loos, Thomas Malinka, Giovanni Marchegiani, Christopher Månsson, Riccardo Pellegrini, Giampaolo Perri, L.H. Poelsler, Geert Roeyen, Michael Rousek, Pablo Sancho, Stina Schild-Suhren, Thomas Schmidt, Leyre Serrablo, Alejandro Serrablo, Andrew Malvern Smith, Gregor A. Stavrou, Oliver Strobel, Shailesh Shrikhande, Alexandra Strobel, Ignazio Tarantino, Jozef Urdzik, Tim Vilz, Roberta Vella, Johanna Wennerblom, Patricia Wyzlic, Jörg Kleeff, and Multivisceral Pancreatic Resections Research Group: A Collaboration of the European-African Hepato-Pancreato-Biliary Association (E-AHPBA)

Collaborative Authors and Affiliations

The following collaborators contributed to this work as members of the Multivisceral Pancreatic Resections Research Group: A Collaboration of the European-African Hepato-Pancreato-Biliary Association (E-AHPBA).

Collaborative Authors

Erik Bergquist[1], Christina Biörserud[2,22], Christiane J. Bruns[3], Thiery Chapelle[4], Olga Ciccarelli[5], Umberto Cillo[6], Omid Ghamarnejad[7], Allison Kinany[8], M Maglione[9], Jiri Molacek[10], Klaudia Narbekovas[11], Melina Neuhaus[21], UP Neumann[12], Radek Pohnán[13], Elizabeth Pando[14], Souheil Reda[15], Amine Sebai[16], Recep Ercin Sonmez[17], Konstantinos Tepetes[18], Lea Timmermann[19], Savvas Tsaramanidis[20], Rizky Widyaningsih[7].

Affiliations

1. Department of Clinical Science, Intervention and Technology (CLINTEC) Karolinska Institutet
2. Region Västra Götaland, Sahlgrenska University Hospital, Dept. of Surgery, Gothenburg, Sweden
3. Faculty of Medicine and University Hospital of Cologne, Department of General, Visceral, Thoracic and Transplantation Surgery, University of Cologne, Cologne, Germany
4. Antwerp University Hospital, Department of Hepatopancreaticobiliary, Endocrine and Transplantation Surgery, Edegem, Belgium
5. Department of Abdominal Surgery and Transplantation at Cliniques Universitaires Saint-Luc, Brussels, Belgium
6. Hepato-pancreato-biliary and Liver Transplant Surgery Unit, Department of Surgical, Oncological and Gastroenterological Sciences (DiSCOG), University of Padua, Padua, Italy
7. Department of General, Visceral and Thoracic Surgery, Surgical Oncology, Klinikum Saarbruecken, Saarbruecken, Germany
8. St James University Hospital, Leeds, UK
9. Department of Visceral, Transplant and Thoracic Surgery, Innsbruck, Austria
10. Department of Surgery, University Hospital in Plzen, Czech Republic
11. Department of General Surgery, Division of Visceral Surgery, Medical University of Vienna, Austria
12. Department of Surgery, Maastricht University Medical Centre, Maastricht, the Netherlands
13. Department of Surgery, Second Faculty of Medicine of Charles University and Military University Hospital, Prague, Czech Republic
14. Pancreatic Surgery Unit, General Surgery and Gastrointestinal Department, Hospital Universitari Vall d'Hebrón, Barcelona, Spain
15. Department of Surgical Sciences, Uppsala University, Uppsala, Sweden
16. Surgery Department A, La Rabta Hospital, La Rabta Jebbari, 1007 Tunis, Tunisia
17. Istanbul Medeniyet University, Goztepe Prof. Dr. Suleyman Yalcin City Hospital, Istanbul, Turkey
18. Department of Surgery, University Hospital of Larissa, Mezourlo, Larissa, Greece

19. Department of Surgery, CCM | CVK, Charité – Universitätsmedizin Berlin, corporate member of Freie Universität Berlin and Humboldt-Universität zu Berlin, Berlin, Germany

20. 5th Surgical Department, Hippocrates General Hospital of Thessaloniki, Greece

21. Department of Visceral, Vascular and Endocrine Surgery, University Hospital Halle (Saale), Martin-Luther-University Halle-Wittenberg, Halle, Germany

22. Dept of Surgery, Sahlgrenska University Hospital, Region Västra Götaland, Gothenburg, Sweden

Supplementary Figure S1. Patient flow diagram.

Flow of patients from the overall registry through PDAC diagnosis, eligibility for propensity score matching (complete covariate data), 1:1 matching, and availability of valid survival data.

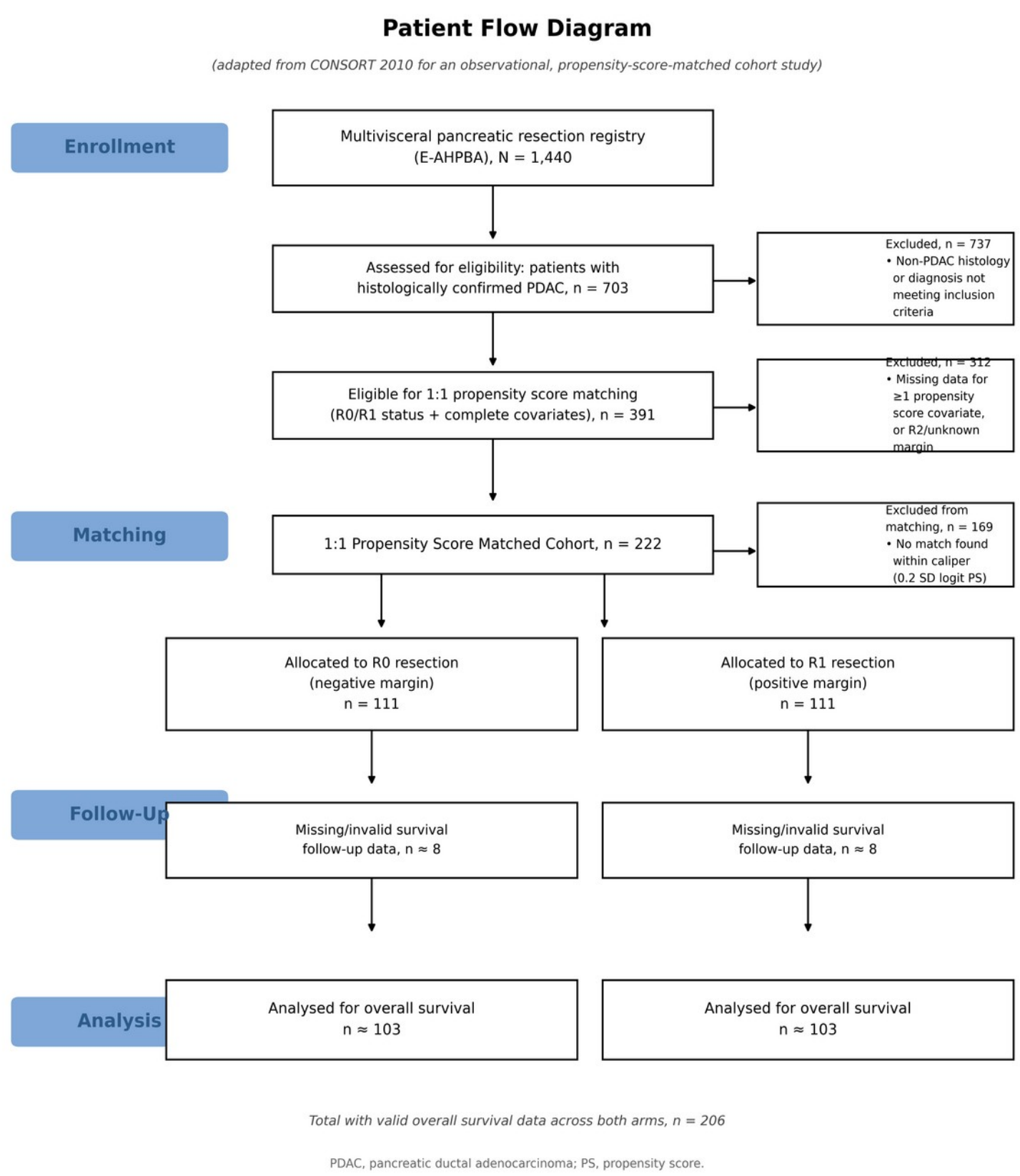

Fig. S1

Balance diagnostic for PSM

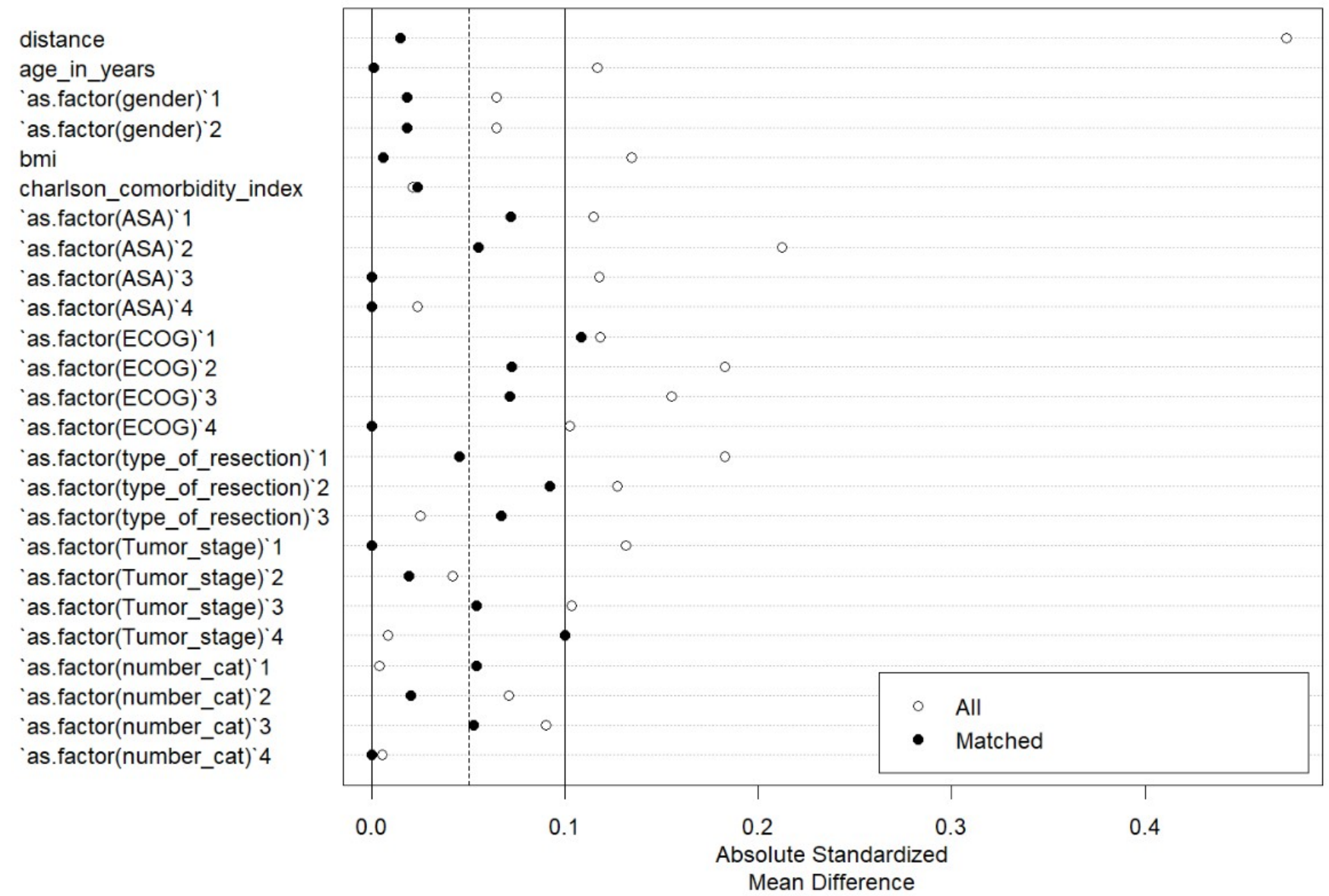

Fig. S2: Love plot showing absolute SMD before and after PS matching

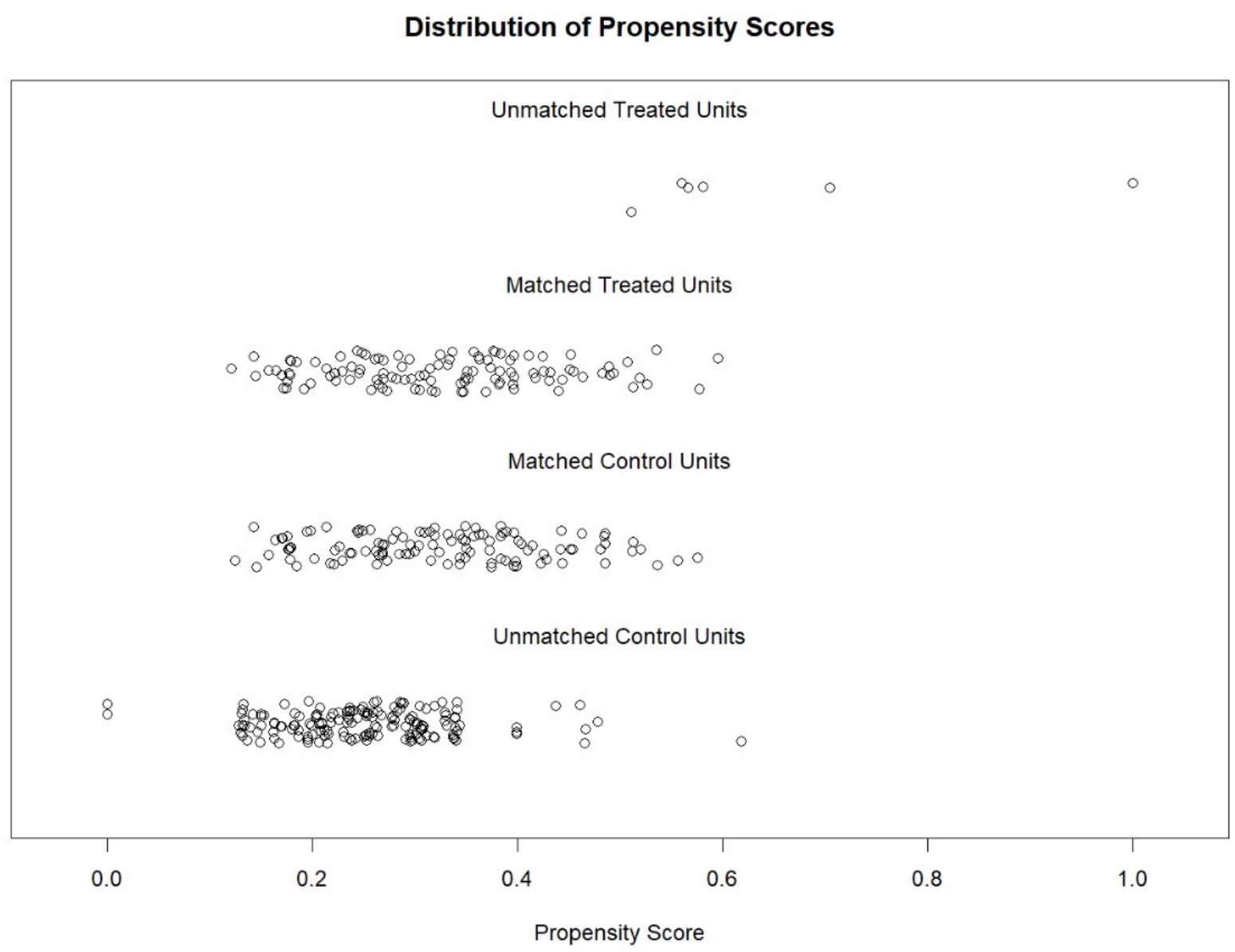

Fig. S3: Jitter plot displaying PS distribution

Tab. S1: z-differences as balance measure before and after PS matching

	Before Matching	After Matching
Age	1.19	-0.04
Gender	0.63	0.13
BMI	-1.19	-0.04
CCI	0.19	-0.19

ASA	-0.44	-0.23
ECOG	0.69	0.89
Type of resection	1.21	-0.12
Tumor stage	-0.79	-0.41
Number of resected organs	0.19	0.43

Sensitivity analysis

Tab. S2: Univariable Cox regression for overall cohort

Characteristic	HR	95% CI	p-value
R0 Resection	Ref.	—	
R1 Resection	1.65	1.29, 2.12	<0.001

Tab. S3: Multivariable Cox regression for overall cohort, adjusted for covariates also used for PSM

Characteristic	HR	95% CI	p-value
R0 Resection	Ref.		
R1 Resection	1.78	1.28, 2.45	<0.001
Age (years)	1.01	1.00, 1.01	<0.001
Female (ref: Male)	0.90	0.68, 1.20	0.5
BMI	0.98	0.95, 1.02	0.3
CCI	1.08	0.93, 1.25	0.3
ASA 2 (ref: 1)	0.89	0.57, 1.38	0.6
ASA 3 (ref: 1)	1.20	0.73, 1.97	0.5
ASA 4 (ref: 1)	2.60	0.94, 7.17	0.065
ECOG 2 (ref: 1)	1.04	0.76, 1.41	0.8
ECOG 3 (ref: 1)	0.93	0.46, 1.89	0.8
DP (ref: TP)	0.89	0.58, 1.36	0.6
PD (ref: TP)	1.34	0.82, 2.17	0.2
Stage II (ref: I)	1.07	0.58, 1.94	0.8
Stage III (ref: I)	1.24	0.69, 2.21	0.5
Stage IV (ref: I)	0.81	0.41, 1.63	0.6
2 organs (ref: 1)	1.24	0.87, 1.77	0.2
3 organs (ref: 1)	1.27	0.78, 2.05	0.3
4+ organs (ref: 1)	0.88	0.45, 1.70	0.7

Tab. S4: Multivariable Cox regression with multiple imputation (MICE, m=20), adjusted for covariates also used for PSM

Characteristic	HR	95% CI	p-value
R0 Resection	Ref.		
R1 Resection	1.56	1.20, 2.04	<0.001
Age (years)	1.01	1.00, 1.01	<0.001
Female (ref: Male)	0.96	0.76, 1.23	0.76
BMI	0.99	0.96, 1.02	0.53
CCI	1.08	0.95, 1.22	0.24
ASA 2 (ref: 1)	0.86	0.58, 1.27	0.45
ASA 3 (ref: 1)	1.20	0.77, 1.86	0.43
ASA 4 (ref: 1)	1.43	0.48, 4.27	0.52
ECOG 2 (ref: 1)	1.06	0.76, 1.41	0.8
ECOG 3 (ref: 1)	0.88	0.46, 1.89	0.8
ECOG 4 (ref: 1)	0.69	0.09, 5.38	0.72
DP (ref: TP)	0.79	0.54, 1.15	0.21
PD (ref: TP)	1.27	0.84, 1.92	0.26
Stage II (ref: I)	1.31	0.78, 2.21	0.31
Stage III (ref: I)	1.51	0.91, 2.49	0.11
Stage IV (ref: I)	1.24	0.69, 2.23	0.48
2 organs (ref: 1)	1.19	0.89, 1.61	0.23
3 organs (ref: 1)	1.07	0.70, 1.64	0.76
4+ organs (ref: 1)	1.06	0.62, 1.82	0.84

Abbreviations: HR, hazard ratio; CI, confidence interval; TP, total pancreatectomy; DP, distal pancreatectomy; PD, pancreatoduodenectomy.
