# Supplementary material for: R0 Resection improves survival after multivisceral resection for pancreatic ductal adenocarcinoma
Source: iScience. 2026 Sep 1;29(9):117352. doi: 10.1016/j.isci.2026.117352 (PMC13571119; doi:10.1016/j.isci.2026.117352)
Supplement: Supplementary file 2 [file mmc2.pdf]

Data S1: Completed STROBE checklist for reporting of this observational cohort study.

STROBE Statement — Checklist of items that should be included in reports of cohort studies

Manuscript: “R0 Multivisceral Resections Are Feasible and Improve Outcomes in Pancreatic Ductal Adenocarcinoma: A Propensity Score-Matched Analysis” (ISCIENCE-D-26-04040)

Section/Topic	Item No	Recommendation	Reported on page/line No.
Title and abstract	1	(a) Indicate the study's design with a commonly used term in the title or the abstract	Title, line 1–2 (“Propensity Score-Matched Analysis”); Abstract/Methods, line 159–160 (“retrospective, international, multicentre cohort study”)
		(b) Provide in the abstract an informative and balanced summary of what was done and what was found	Summary (Background/Methods/Findings/Interpretation), lines 145–202
**Introduction**			
Background/rationale	2	Explain the scientific background and rationale for the investigation being reported	Introduction, lines 210–232
Objectives	3	State specific objectives, including any prespecified hypotheses	Introduction, lines 233–240
**Methods**			
Study design	4	Present key elements of study design early in the paper	Methods – Study Design and Setting, line 246–251; Statistical Analysis (propensity score matching), lines 304–312
Setting	5	Describe the setting, locations, and relevant dates, including periods of recruitment, exposure, follow-up, and data collection	Methods – Study Design and Setting, lines 246–258 (Jan 2010–Dec 2022; ethics approval, processing number 2023-211)
Participants	6	(a) Give the eligibility criteria, and the sources and methods of selection of participants.	Methods – Participants, lines 260–274 (Inclusion/Exclusion Criteria)

Section/Topic	Item No	Recommendation	Reported on page/line No.
		Describe methods of follow-up	
		(b) For matched studies, give matching criteria and number of exposed and unexposed	Statistical Analysis, lines 305–312 (1:1 nearest-neighbour PS matching, covariates); Results, lines 339–360 and Table 2 (n=391 pre-match, n=222 post-match, 111 R0/111 R1)
Variables	7	Clearly define all outcomes, exposures, predictors, potential confounders, and effect modifiers. Give diagnostic criteria, if applicable	Methods – Intervention/Exposure and Outcomes, lines 276–300 (Refs 6–11); Statistical Analysis, lines 308–312 (PS covariates)
Data sources/measurement	8*	For each variable of interest, give sources of data and details of methods of assessment (measurement). Describe comparability of assessment methods if there is more than one group	Methods – Study Design and Setting, lines 249–251 (centralised REDCap database, standardized collection); Outcomes, lines 287–300 (Clavien–Dindo, ISGPS, Satava, Royal College of Pathologists definitions)
Bias	9	Describe any efforts to address potential sources of bias	Statistical Analysis, lines 305–323 (propensity score matching, adjustment for residual imbalance); Limitations of the Study, lines 497–510 (items 1–3: selection bias, missing data, residual confounding)
Study size	10	Explain how the study size was arrived at	Results, lines 339–344 (1,440 patients overall, 703 PDAC; 391 eligible for matching; 222 matched)
Quantitative variables	11	Explain how quantitative variables were handled in the analyses. If applicable, describe which groupings were chosen and why	Statistical Analysis, lines 304–312; Table 1/Table 2
Statistical methods	12	(a) Describe all statistical methods, including those used to control for confounding	Statistical Analysis, lines 304–323
		(b) Describe any methods used	Statistical Analysis, lines 319–323 (complete-

Section/Topic	Item No	Recommendation	Reported on page/line No.
		to examine subgroups and interactions	cohort Cox regression as sensitivity analysis)
		(c) Explain how missing data were addressed	Statistical Analysis, lines 325–331 (multiple imputation by chained equations, m=20); Limitations of the Study, lines 500–506 (item 2)
		(d) If applicable, explain how loss to follow-up was addressed	Not applicable – retrospective registry-based cohort; overall survival censored at last available follow-up (Statistical Analysis, lines 316–318)
		(e) Describe any sensitivity analyses	Statistical Analysis, lines 319–332 (complete-case Cox regression and multiple-imputation sensitivity analysis; results consistent, lines 388–390 and Supplementary Tables S2–S4)
**Results**			
Participants	13*	(a) Report numbers of individuals at each stage of study—eg numbers potentially eligible, examined for eligibility, confirmed eligible, included in the study, completing follow-up, and analysed	Results, lines 339–344; CONSORT-style flow diagram, Supplementary Figure S0
		(b) Give reasons for non-participation at each stage	Results, lines 341–344 (exclusion due to incomplete covariate data for propensity score matching); Limitations of the Study, lines 500–506
		(c) Consider use of a flow diagram	Supplementary Figure S0 (CONSORT-style flow diagram)
Descriptive data	14*	(a) Give characteristics of study participants (eg demographic, clinical, social) and information on exposures and potential confounders	Table 1 (overall cohort) and Table 2 (matched cohort), referenced at lines 339–360

Section/Topic	Item No	Recommendation	Reported on page/line No.
		(b) Indicate number of participants with missing data for each variable of interest	Table 1 footnotes (missingness by variable); Results, lines 341–344; Limitations of the Study, lines 500–506
		(c) Summarise follow-up time (eg, average and total amount)	Results, lines 373–379 (median overall survival and 1-, 3-, 5-year survival rates as follow-up summary)
Outcome data	15*	Report numbers of outcome events or summary measures over time	Results, lines 361–379 and Tables 3–4 (perioperative outcomes and survival)
Main results	16	(a) Give unadjusted estimates and, if applicable, confounder-adjusted estimates and their precision (eg, 95% confidence interval). Make clear which confounders were adjusted for and why they were included	Results, lines 382–388 and Tables 5–6 (univariable and multivariable Cox regression, HR with 95% CI)
		(b) Report category boundaries when continuous variables were categorized	Table 1/Table 2 (age, BMI, CCI, ASA, ECOG categories)
		(c) If relevant, consider translating estimates of relative risk into absolute risk for a meaningful time period	Results, lines 376–379 (1-, 3-, and 5-year survival rates for R0 vs R1)
Other analyses	17	Report other analyses done—eg analyses of subgroups and interactions, and sensitivity analyses	Results, lines 388–390; Supplementary Tables S2–S4 (complete-case and multiple-imputation sensitivity analyses)
**Discussion**			
Key results	18	Summarise key results with reference to study objectives	Discussion, lines 395–417 (opening summary paragraphs)
Limitations	19	Discuss limitations of the study, taking into account sources of potential bias or	Limitations of the Study, lines 495–536

Section/Topic	Item No	Recommendation	Reported on page/line No.
		imprecision. Discuss both direction and magnitude of any potential bias	
Interpretation	20	Give a cautious overall interpretation of results considering objectives, limitations, multiplicity of analyses, results from similar studies, and other relevant evidence	Discussion, lines 454–465 (cautious/associative rather than causal interpretation); Conclusions, lines 538–546
Generalisability	21	Discuss the generalisability (external validity) of the study results	Limitations of the Study, lines 511–528 (items 4 and 7–8: heterogeneity across centres, absence of formal LAPC/borderline-resectable classification, variability in systemic therapy over time)
**Other information**			
Funding	22	Give the source of funding and the role of the funders for the present study and, if applicable, for the original study on which the present article is based	Funding statement, line 596 (“This research received no external funding”)

**Give information separately for exposed and unexposed groups (R0 vs R1).*

Note: An Explanation and Elaboration article discusses each checklist item and gives methodological background and published examples of transparent reporting. The STROBE checklist is best used in conjunction with this article (freely available on the websites of PLoS Medicine at www.plosmedicine.org, Annals of Internal Medicine at www.annals.org, and Epidemiology at www.epidem.com). Information on the STROBE Initiative is available at www.strobe-statement.org.
